# Association between Polymorphisms in Vitamin D Pathway-Related Genes, Vitamin D Status, Muscle Mass and Function: A Systematic Review

**DOI:** 10.3390/nu13093109

**Published:** 2021-09-04

**Authors:** Ermira Krasniqi, Arben Boshnjaku, Karl-Heinz Wagner, Barbara Wessner

**Affiliations:** 1 Research Platform Active Ageing, University of Vienna, Althanstraße 14, 1090 Vienna, Austria; ph.ermirakrasniqi@gmail.com (E.K.); karl-heinz.wagner@univie.ac.at (K.-H.W.); 2 Centre for Sport Science and University Sports, University of Vienna, Auf der Schmelz 6, 1150 Vienna, Austria; arbenboshnjaku@gmail.com; 3 Department of Nutritional Sciences, University of Vienna, Althanstraße 14, 1090 Vienna, Austria; 4 Faculty of Medicine, University “Fehmi Agani” in Gjakova, Ismail Qemali n.n., 50000 Gjakovë, Kosovo

**Keywords:** vitamin D, genetic variations, SNPs, GC, CYP2R1, VDR, CYP24A1, muscle-related traits

## Abstract

An association between vitamin D level and muscle-related traits has been frequently reported. Vitamin D level is dependent on various factors such as sunlight exposure and nutrition. But also on genetic factors. We, therefore, hypothesize that single nucleotide polymorphisms (SNPs) within the vitamin D pathway-related genes could contribute to muscle mass and function via an impact on vitamin D level. However, the integration of studies investigating these issues is still missing. Therefore, this review aimed to systematically identify and summarize the available evidence on the association between SNPs within vitamin D pathway-related genes and vitamin D status as well as various muscle traits in healthy adults. The review has been registered on PROSPERO and was conducted following PRISMA guidelines. In total, 77 studies investigating 497 SNPs in 13 different genes were included, with significant associations being reported for 59 different SNPs. Variations in GC, CYP2R1, VDR, and CYP24A1 genes were reported most frequently, whereby especially SNPs in the GC (rs2282679, rs4588, rs1155563, rs7041) and CYP2R1 genes (rs10741657, rs10766197, rs2060793) were confirmed to be associated with vitamin D level in more than 50% of the respective studies. Various muscle traits have been investigated only in relation to four different vitamin D receptor (VDR) polymorphisms (rs7975232, rs2228570, rs1544410, and rs731236). Interestingly, all of them showed only very low confirmation rates (6–17% of the studies). In conclusion, this systematic review presents one of the most comprehensive updates of the association of SNPs in vitamin D pathway-related genes with vitamin D status and muscle traits in healthy adults. It might be used for selecting candidate SNPs for further studies, but also for personalized strategies in identifying individuals at risk for vitamin D deficiency and eventually for determining a potential response to vitamin D supplementation.

## 1. Introduction

In recent years, the association between “optimal” serum levels of vitamin D with different healthcare conditions has been given important attention in medical research. Studies have shown that various factors such as season, latitude (ultraviolet B (UVB) availability), air pollution, clothing style, sunshine exposure, skin pigmentation, sunscreen cream, age, diet, and nutritional supplementation directly or indirectly affect vitamin D status [1,2,3].

In the body, vitamin D occurs in different forms with several enzymes being involved in their metabolism. Briefly, vitamin D2 (ergocalciferol) built from the provitamin ergosterol and vitamin D3 (cholecalciferol) originating from 7-dehydrocholesterol (7-DHC) are converted into the circulating 25-hydroxyvitamin D (including 25(OH)D2 and 25(OH)D3) and the biologically active 1,25-hydroxy-cholecalciferol (including 1,25(OH)2D2 and 1,25(OH)2D3) [4]. Subsequently, the active form exerts its action on various cell types through a specific vitamin D receptor (VDR) [5,6]. The processes including metabolism, transportation and signaling of vitamin D are regulated by a number of proteins encoded by specific genes (i.e., 7-dehydrocholesterol reductase (DHCR7/), cytochrome P450-2R1 (CYP2R1), cytochrome P450-27B1 (CYP27B1), vitamin binding protein (GC/DBP), VDR, and retinoid-X receptor A (RXRA) [7].

Low levels of vitamin D have been reported across various geographical regions [8,9,10]. Interestingly, vitamin D deficiency is commonly detected among older adults and is strongly associated with a decline in physical performance in this age group particularly [11]. Additionally, vitamin D deficiency has been described to be among the factors that lead to sarcopenia [5,12], a progressive, generalized, and age-related skeletal muscle disorder [13]. Furthermore, the supplementation of vitamin D seems to ameliorate mobility and muscle strength [14,15], especially when combined with resistance training [16], but also enhance muscle performance [17,18], suggesting a direct connection between vitamin D and muscle function. One compelling review from Garcia and colleagues described most appropriately the need to assess vitamin D level as one way to minimize physiological and functional changes in skeletal muscle [5].

Besides environmental factors influencing vitamin D levels, a genetic impact has also been questioned in various studies [19,20], and some of them investigated whether single nucleotide polymorphisms (SNPs) in vitamin D-associated genes such as the VDR contribute to muscular strength and mass [21]. However, investigation and integration of studies that explored the association of vitamin D pathway-related genes with muscle traits other than VDR are still missing to date. 

In an attempt to broaden this hypothesis, we further assume that genetic variants in several vitamin D pathway-related genes would affect vitamin D levels and, as a consequence, various parameters of physical performance. Therefore, the aim of this systematic review was to comprehensively identify published SNPs in genes known to be associated with the vitamin D pathway and then to systematically analyze their influence on vitamin D levels and/or muscle function in adults, including older adults.

## 2. Materials and Methods

Details of the protocol for this systematic review were registered on PROSPERO [22]. The report followed the PRISMA (Preferred Reporting Items for Systematic Reviews and Meta-Analyses) guidelines [23].

### 2.1. Search Strategy 

**Figure 1:**
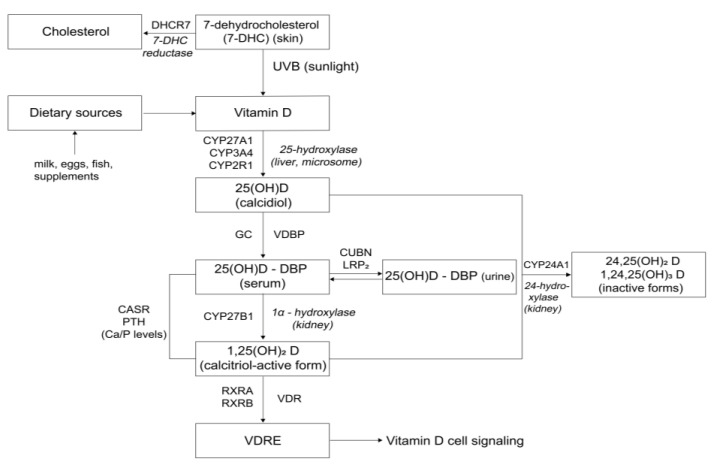
Vitamin D pathway, candidate genes (in bold), and associated enzymes. DHCR7 (7-dehydrocholesterol) gene encodes 7-DHC (7-dehydrocholesterol) reductase enzyme, which converts 7-DHC to cholesterol; CYP2R1 (cytochrome P450 family 2 subfamily R member 1), CYP3A4 (cytochrome P450 family 3 subfamily A member 4), and CYP27A1 (cytochrome P450 family 27 subfamily A member 1) genes encode 25-hydroxylation cytochrome P450 enzymes responsible for converting provitamin D that is absorbed from the diet or synthesized from the action of sunlight on the skin to the circulating form 25(OH)D (25-hydroxyvitamin D); vitamin D is transported bound to vitamin D binding protein (DBP) (encoded by GC gene); LRP2 and CUBN genes encode plasma membrane receptors megalin and cubilin, respectively (involved in re-absorption of 25(OH)D via receptor mediated endocytosis); CYP27B1 encodes the cytochrome p450 enzyme which coverts 1-alpha-hydroxylates 25(OH)D to the active form 1,25(OH)_2_D (1,25-Dihydroxycholecalciferol, Calcitriol); CASR (calcium sensing receptor) binds calcium in extracellular matrix, impacting calcium homeostasis; Ca homeostasis impacts the synthesis of parathyroid hormone (PTH gene-a protein coding gene) which stimulates the synthesis of 1,25(OH)_2_D from 25(OH)D by upregulating renal 1-α-hydroxylase; CYP24A1 encodes a 24-hydroxylase enzyme which catalyzes the degradation of 25(OH)D and 1,25(OH)_2_D in inactive metabolites; VDR encodes the vitamin D receptor, a nuclear receptor which binds 1,25(OH)_2_D and forms a heterodimer with the gene product of RXR—the retinoid X receptor—to mediate the biological actions of vitamin D.

In order to identify potential candidate genes, PathCards (https://pathcards.genecards.org/, accessed on 27 November 2018) was used to identify genes that are related to the vitamin D pathway (*n* = 10). PathCards is a constituent network of metabolic pathways with mapping genes, which provides researchers with a rich, searchable systems analysis resource [24]. Additionally, we searched for similar genetic association studies to identify other candidate genes encoding key player proteins (LRP2, CUBN, CYP3A4, and CASR (calcium-sensing receptor)) [19,20]. Information on the finally identified 14 genes is summarized in Figure 1. In order to find publications related to genetic variations in each of the genes, the respective NCBI entry in the gene database was linked first to the SNP database of each gene and then to the PubMed entries. Consequently, these results were connected to a conservative search strategy on PubMed using the respective gene name or their aliases connected with the MeSH term “genetic variation”.

The final search for PubMed was conducted on 27 November 2018 and followed a protocol developed a priori. The search strategy aimed to identify all articles where the specific genetic variants (i) were aligned with search terms for either vitamin D status (ii), muscle traits (iii), or both (ii AND iii):

(i) each of the 14 vitamin D-pathway-related genes (including their aliases variation (MeSH terms])
(ii) vitamin D status (vitamin D[MeSH] OR 25(OH)D OR 25(OH)D2 OR 25(OH) D3 OR Vitamin D Deficiency[MeSH] OR “vitamin D status” OR “vitamin D level” OR “vitamin D inadequacy” OR “hypovitaminosis D” OR “avitaminosis D”) NOT Review[Publication Type], Filters: Humans; English; German
(iii) muscle strength and function (Muscle, Skeletal[MeSH] OR Muscle Strength[MeSH] OR Physical Fitness[MeSH] OR Walking Speed[MeSH] OR “muscle mass” OR “lean body mass” OR “muscle quality” OR “physical performance” OR SPPB OR “short physical performance battery” OR “handgrip strength” OR “chair stand” OR “arm curl”) NOT Review[Publication Type], Filters: Humans; English; German

After conducting the initial structured search as outlined above, additional studies were added based on the reference lists of the finally selected studies (hand search).

### 2.2. Inclusion and Exclusion Criteria

Articles were included if studies:

(i) were conducted among humans aged >18 years from both sexes;
(ii) comprised candidate or genome-wide association studies being cross-sectional, cohort, case control, or intervention studies;
(iii) investigated healthy subjects or contained at least a separate control group with normal health status;
(iv) measured at least one genotype in a vitamin D pathway-related gene;
(v) reported vitamin D status (circulating plasma/serum levels, 25(OH)D, 25(OH)D2, or 25(OH)D3 metabolites) and/or reported results for muscle mass or function, muscle strength parameters or scores for the Short Physical Performance Battery (SPPB);
(vi) were published in English or German.

Articles were excluded if they:

(i) reported only vitamin D intake or vitamin D metabolites from urine;
(ii) investigated children (<18 years old);
(iii) investigated participants with severe chronic or acute illnesses with a known impact on either vitamin D status and/or muscle mass and strength (myopathies, hypocalciuric hypercalcemia), or pregnant or lactating women;
(iv) were published as case reports, systematic reviews, or meta-analyses.

### 2.3. Study Selection and Data Extraction

Study selection and data extraction was performed by two independent reviewers (EK, BW) in accordance with the above-mentioned inclusion and exclusion criteria. A third reviewer (KHW) was included in case of disagreements.

Data extraction from eligible studies included information as follows: (i) gene, (ii) SNP, (iii) bibliographic information, (iv) study design, (v) participants (age, gender, and ethnicity), (vi) sample size, (vii) main findings and outcomes, (viii) location, sampling season and analysis method of vitamin D level if available.

A narrative synthesis of the findings from the selected studies was performed using two frameworks in accordance with the following specific research questions: (i) association of vitamin D-related genetic polymorphisms and vitamin D status, and (ii) association of vitamin D-related genetic polymorphisms and muscle mass or strength. Results were described qualitatively rather than performing a quantitative meta-analysis as study designs and reported outcomes differed widely in order to quantitatively analyze them. 

### 2.4. Risk of Bias (Quality) Assessment

In order to assess the quality of included studies, the STREGA recommendations (STrengthening the REporting of Genetic Association studies guidelines) were applied independently by two reviewers (three if there was any disagreement even after discussion) [25]. The quality of the studies was considered as “high” when the score was 18–22, “moderate-high” when the score was 13–17, and “low” with a score below 12. Results were reported, but no restrictions were made with respect to the inclusion or exclusion of the relevant studies. 

## 3. Results

### 3.1. Study Selection and Characteristics

**Figure 2:**
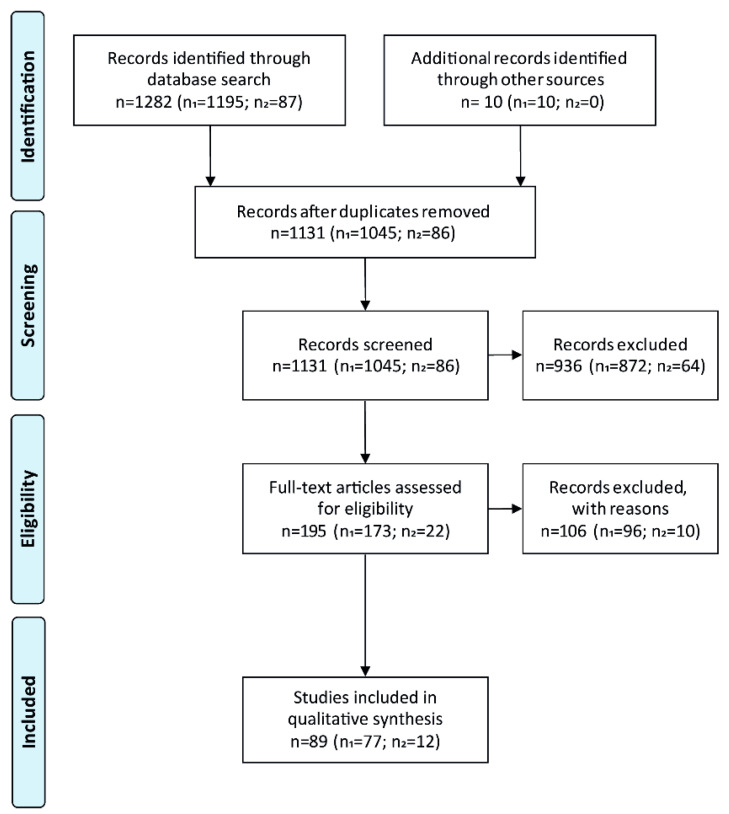
PRISMA-Flow diagram showing the selection of studies included in the systematic review. The number of studies reporting genetic variants and vitamin D status as well as the number of studies reporting genetic variants together with muscle mass and function, are given in parenthesis (n_1_ and n_2_).

In total, 1292 studies were identified from the initial searches in PubMed (*n* = 1282) and through other sources (*n* = 10, Figure 2). As the searches were conducted separately for each of the 14 vitamin D pathway-related genes and then combined, 161 articles had to be excluded as duplicates. During title/abstract screening, a further 936 articles were excluded based on the predefined inclusion and exclusion criteria. The remaining 195 articles were assessed for eligibility based on the full texts. Among these, a further 106 studies were not suitable with respect to the research question. Consequently, 89 studies were included in the systematic review, with 77 of them reporting the association of genetic polymorphisms of vitamin D-related genes and vitamin D status, and only 12 studies dealing with genetic variants of vitamin D-related genes and muscle mass and/or function. Study designs included 57 cross-sectional studies, 20 case-control studies, and 12 intervention studies. 

### 3.2. Polymorphisms in Vitamin D Pathway-Related Genes and Vitamin D Status 

As summarized in Table 1, 77 publications that have investigated a potential association between a certain genetic association and vitamin D levels were included in the report. Most of these publications were cross-sectional studies (*n* = 46); a further 19 studies were conducted as case-control studies and 12 studies as interventions. Altogether, these studies investigated 497 SNPs in 13 different genes (GC, CYP2R1, VDR, CYP24A1, DHCR7, CYP27B1, CYP27A1, CASR, PTH, CYP3A4, RXRA, CUBN, and RXRB). No suitable records were found for the gene lipoprotein receptor-related protein 2 (megalin, LRP2).

The publication date of the included papers ranged from 2002 to 2018. In total, 81,896 healthy participants were investigated, whereby the number of study participants ranged from 31 (case-control) [89] to 8417 (cross-sectional studies) [57]. Participants with certain diseases such as type 1 [33] and type 2 [83] diabetes mellitus, osteomalacia [89], COPD [45], coronary artery disease [64], hemodialysis [87], Crohn’s disease [86], pulmonary tuberculosis [100], melanoma [34], prostate [26,42], breast [32,61,73,97], colorectal [69] and non-small cell lung cancer [75] comprised the cases in the included case-control studies. However, the results of these participants were not included in the analyses of this systematic review.

The most frequently studied gene comprised the vitamin D binding protein (GC) which was investigated in 56 studies, followed by CYP2R1, coding for a vitamin D 25-hydroxylase, which was mentioned in 41 studies, and the vitamin D receptor (VDR) having been subject to 41 studies. In total, 59 SNPs located within 10 different genes showed a significant association with vitamin D levels in at least one study. Most importantly, 23 of these SNPs were confirmed to be related to vitamin D status in at least two other studies (Table 1). For genetic variants in the CYP27A1 gene (vitamin D 25-hydroxylase), CUBN gene (cubilin), and RXRB gene (retinoid-X receptor B), none of the studies reported a significant association with vitamin D level [26,27,30,33,71,101]. 

SNPs that were studied in at least 15 different studies showed significant associations in 8–77% of the respective studies. The highest confirmation rates were found for SNPs in the GC gene [rs2282679 (association to vitamin D status confirmed in 23 out of 30 studies (77%)); rs4588 (confirmed in 27 out of 37 studies (73%)); rs1155563 (confirmed in 12 out of 17 studies (71%)); rs7041 (confirmed in 27 out of 39 studies (69%))] and in the CYP2R1 gene [rs10741657 (confirmed in 21 out of 32 studies (66%)); rs10766197 (confirmed in 9 out of 15 studies (60%)); rs2060793 (confirmed in 8 out of 15 studies (53%))]. Further frequently studied SNPs located in the DHCR7 gene [rs12785878, confirmed in 6 out of 19 studies (32%)], the CYP24A1 gene [rs6013897, confirmed in 3 out of 18 studies (17%)], and the CYP2R1 gene [rs10877012, confirmed in 2 out of 15 studies (13%)]. Interestingly, SNPs in the VDR gene were frequently investigated, but their confirmation rate was very low [rs7975232 (alias ApaI, confirmed in 3 out of 18 studies (17%)); rs2228570 (confirmed in 4 out of 25 studies (16%)); rs1544410 (confirmed in 4 out of 28 studies (14%)); and rs731236 (confirmed in 2 out of 24 studies (8%)); rs11568820 (confirmed in 1 out of 16 studies (6%))]. A complete list can be found in Supplementary Table S1).

Most of the studies were performed in Europe (21 studies from Belgium, Czech Republic, Denmark, Finland, France, Germany, Hungary, Netherlands, Norway, Estonia, UK, France, Italy, Greece, Spain, Scotland, Sweden, United Kingdom), followed by the US (14 studies) and China (11 studies). 

Measurement methods for vitamin D levels were very diverse and included radioimmunoassay, enzyme-linked immunosorbent assay (ELISA), chemiluminescent immunoassays, high performance liquid chromatography (HPLC), and liquid chromatography–tandem mass spectrometry. Mostly, total 25(OH)D (summing up 25(OH)D2 and 25(OH)D3) was measured, although some studies [20,26,34,37,39,41,42,49,52,54,56,70,76,83,92,94,95,100] discriminated between different vitamin D metabolites (Supplementary Table S1). 

### 3.3. Polymorphisms in Vitamin D Pathway-Related Genes and Muscle Mass and Function

As reported in Table 2, 12 publications were included in the qualitative synthesis, whereby 11 studies were conducted as cross-sectional studies and one study as a case-control study [103]. All the selected studies were focusing on potential associations between VDR gene polymorphisms and muscle traits, investigating only four different SNPs in this gene [rs7975232 (alias ApaI), rs1544410 (alias BsmI), rs2228570 (alias FokI, including the merged SNP rs10735810), and rs731236 (alias TaqI)].

In total, 5342 healthy subjects were included, with the number of participants ranging from 104 (case-control study) [103] to 1970 (cross-sectional study) [110]. The most frequently studied SNP was rs1544410 (BsmI), investigated by 11 studies, whereby five studies were reporting significant associations between its genotypes (BB, Bb, bb) and muscle traits such as knee flexion peak torque [104], knee extensor strength [109], maximal power [110], hamstring strength [112] and quadriceps strength [114]. Four studies included only female subjects [104,110,112,114]. 

The rs2228570 (FokI, rs10735810) SNP was mentioned in eight studies, five of which showing a significant association between its genotypes (FF, Ff, ff) and muscle traits: quadriceps strength [103,108,113], handgrip strength [106], and knee extension strength [111]. 

Rs7975232 (ApaI) was investigated by three studies [104,105,106], one of them showing significance between aa + aA genotypes and muscle strength [104]. For the genotypes of the SNP rs731236 (TaqI), none out of five studies reported any significant association [104,105,106,108,109].

### 3.4. Quality of Included Studies

The STREGA quality score for the studies relating the respective SNPs to vitamin D status was 18.8 ± 2.3 showing low to high quality with a range between 11 and 22. While for studies relating SNPs to muscle traits, the mean STREGA score was 16.8 ± 1.8 with a range between 13 and 19, indicating moderate to high quality. 

## 4. Discussion

We have systematically evaluated the available scientific data showing the association of certain genotypes to vitamin D deficiency, and hence, poor muscle status. Variations in GC, CYP2R1, VDR, and CYP24A1 genes were reported most frequently, whereby especially SNPs in the GC (rs2282679, rs4588, rs1155563, rs7041) and CYP2R1 genes (rs10741657, rs10766197, rs2060793) were confirmed to be associated with vitamin D plasma level in more than 50% of the respective studies. Various muscle traits have been investigated only in relation to four different VDR polymorphisms (rs7975232, rs2228570, rs1544410, and rs731236). Interestingly, all of them showed only very low confirmation rates (6–17% of the studies).

Synthesized or consumed with the diet, in the liver, vitamin D is converted in its circulating form 25-hydroxyvitamin D (calcidiol), a process mediated by enzyme 25-hydroxylase, which is encoded by the CYP2R1 gene. Polymorphisms in this gene may impact vitamin D metabolism, while it shows catabolic effects toward Vitamin D2 and D3 by modulating 25-hydroxylase’s activity and expression [115]. Accordingly, a significant impact of 9 polymorphisms on this gene (Table 1) with vitamin D status has been found to be replicated in more than two studies. Rs10741657, located in non-coding region 5′-UTR, was the most frequently studied SNP of this gene, with a significant association confirmed in 66% of the conducted studies with the beneficiary allele being the minor one. This data is in line with a recent meta-analysis on the effects of CYP2R1 gene variants on vitamin D levels [116]. Being located in 2-kb CYP2R1 mRNA transcript, it is believed that this SNP is able to change enzyme activities and affect vitamin D metabolism [117]. At the same time, two other SNPs, rs12794714 and rs10766197, located in the coding region of introns with the possibility of altering transcription rate [117] are confirmed in 79 and 60%, respectively, with the minor allele being the risk allele in the two aforementioned SNPs (Supplementary Table S1). 

The transport of 25(OH)D toward target tissues for utilization and processes is enabled by vitamin D binding protein (DPB). This protein binds 85 to 90% of vitamin D circulating form, having the role of both carrier and reservoir. The remaining 10–15% of the circulating vitamin D is bound to albumin, or unbound, representing the available fraction based on the free hormone hypothesis [97]. This multifunctional and polymorphic protein is encoded by the GC gene (located on chromosome 4q12-q13), whose DNA sequence may impact the binding ability to vitamin D since its isoforms have different binding affinities, possibly impacting the half-life of circulating 25(OH)D [28]. From at least 120 identified isoforms, the most common ones Gc1f and Gc1s (rs7041 locus), as well as Gc2 (rs4588 locus), refer to the two functional SNPs in exon 11 with rs7041 causing an Asp→Glu amino acid change and rs4588 causing a Thr→Lys exchange in the vitamin D binding protein [118]. Interestingly, those two SNPs were found to be significantly associated with the vitamin D level in 69 and 73% of the respective studies included in this review. In the aforementioned SNPs, the major allele was the beneficiary one in the majority of the studies (70 and 93%, respectively). In addition, this gene’s polymorphisms are believed to influence the circulating concentration of DBP, which may alter the bioavailable circulating vitamin D [119]. This association might also be linked to the possible impact of these SNPs in the affinity of DPB to actin while modifying its actin-binding region and affecting 25(OH)D uptake and retention into skeletal muscle cells. Furthermore, it has been shown that C2 myotubes and primary rat muscle fibers express megalin and cubilin receptors, which enable endocytosis of DBP [120]. Studies on megalin and cubilin published after the search closing date of this systematic review did not reveal new findings [121,122]. In addition to the previously mentioned functional polymorphisms in the GC gene, there are also intron-located SNPs, which were found to be associated with vitamin D status, whereas the underlying mechanism remains unclear. One such, rs2282679, an intron variant (in linkage disequilibrium with rs4588) [123], was found to be significantly associated with vitamin D in 77% of the identified studies, all confirming the major allele as the beneficiary one. Finally, rs1155563 (an intron variant) was confirmed in 71% of the studies (of which 71% confirmed the major allele as the beneficiary one).

Furthermore, another important part of the vitamin D pathway chain is the vitamin D receptor, a high-affinity nuclear receptor encoded by the VDR gene. Vitamin D exerts its biological roles when its active metabolite 1alpha,25-dihydroxyvitamin D3 [1alpha,25(OH)2D3] binds to VDR, causing a transactivation function of VDR [124]. The resulting complex 1,25D-VDR-RXR then binds to vitamin D response elements in the DNA [125]. Consequently, VDR is involved in the regulation of many cellular functions such as phospholipid metabolism, apoptosis, cell differentiation, and oxidative stress. It also affects the expression of the vitamin D metabolism-related genes CYP27B1 and CYP24A1 [126,127]. Several studies aimed to prove the association between VDR gene polymorphism and vitamin D status. Among one of the most studied SNPs, Fok1 is a polymorphism located at the start codon of the coding part, whose polymorphic form produces a protein shorter by amino acids [128], altering the length of the VDR [106]. However, an association with vitamin D level has been confirmed in only 16% of the studies included in this systematic review. The exact role of other frequently studied polymorphisms in VDR is not fully elucidated. Three SNPs located in the 3′ end of the VDR gene rs1544410 (Bsm1), rs731236 (Taq1), and rs7975232 (Apa1), considered to be in high linkage disequilibrium with 3′ UTR polymorphisms [129]. An effective role of these genetic variants on vitamin D level is very unlikely as they have been associated with the vitamin D level in only 14, 8, and 17% of the studies, although investigated in 28, 24, and 18 studies, respectively. Although the regions around these SNPs are not translated to the VDR protein, they might have a role in mRNA stability because of their neighborhood to the poly-A tail [130]. 

Taken together, there seems to be limited evidence that genetic variations in the VDR gene will exert a meaningful association with vitamin D level, given the small number of studies showing significant associations in relation to the high number of studies investigating a potential association. This is further supported by the notation that even in those studies showing a significant association, there was no clear direction with respect to the identification of a beneficiary allele (Supporting File S1).

In addition, two SNPs on the DHCR7 genes have been highly investigated. This gene encodes the enzyme 7-dehydrocholesterol reductase, a key metabolite enzyme that catalyzes the conversion of 7-dehydrocholesterol to cholesterol [28]. Rs12785878 and rs1790349 were found to be significantly associated with the vitamin D level in 32 and 50% of the included studies, while the major allele could be identified to be the beneficiary one in 83 and 80% of the studies. Therefore, no clear conclusion can be drawn on the involvement of these SNPs in affecting vitamin D level, which is confirmed in recent studies on adolescents with rs12785878 genotype showing no association to hypovitaminosis D [131], but an interaction between 25(OH)D levels and rs12785878 genotype in DHCR7 on overall survival of patients with metastatic colorectal cancer [132]. 

While vitamin D deficiency is an important public health topic, at least some vitamin D-related gene polymorphisms seem to play an important role in vitamin D status. However, an in-depth analysis of the study characteristics revealed (see Supplementary Table S1) that the included studies were characterized by heterogeneous methodology, including varying sample sizes, age groups, and, most importantly, different vitamin D measurement techniques. After concerns were raised about the accuracy of different vitamin D assays and the possibilities of misleading assessment of vitamin D levels [133], EFSA recommended liquid chromatography-tandem mass spectroscopy (LC-MS/MS) as the reference method in regard to 25(OH)D concentrations [4]. Notwithstanding, LC-MS/MS was found to be used in only 17 out of 77 studies (22.1%) included in this qualitative analysis, while the radioimmunoassay method (RIA) was the most used one (in 22 of 77 studies, 28.6%). It also must be noted that the search for genotypes differed as well—as most of the studies used a candidate genotype approach, although some SNPs were identified via SNP arrays [26,27,28,30,37,38,52,60,62,65,74,80,81,97]. 

As vitamin D level has been shown to be low in a significant proportion of adults worldwide [134], vitamin D supplementation remains an important method to achieve optimal levels. Its ability to enhance muscle strength [135], physical performance [17,18], including the lowering of the risk of falling in older adults with low serum 25(OH)D [17,136], has already been demonstrated. Notwithstanding, genetics might also affect the metabolic response toward vitamin D supplementation [80]. Accordingly, existing data implicate that polymorphisms in CYP2R1 (rs10766197, rs10741657), GC (rs4588, rs7041, rs2282679,) and VDR gene (rs2228570,) are associated with vitamin D dose-response, in view of the fact that these were individually replicated in at least two different intervention studies [27,35,40,41,54,56,60,74,78,80]. Although this was not the primary aim of this systematic review, it might comprise important information towards the necessity of personalized vitamin D treatment due to a possible intra-individual variability. It should be noted that we have not encountered studies investigating the direct link between vitamin D pathway-related gene polymorphisms and the effect of vitamin D supplementation on muscle traits. Although, data supporting the impact of these genes’ polymorphisms in vitamin D status implicates their potential effect of vitamin D status in health outcomes such as muscle performance, particularly in vitamin D supplementation improvement in older adults with 25(OH)D levels <37–45 nmol/L [17,18].

While some outcomes of vitamin D deficiency, such as osteomalacia and osteoporosis are well known, the implications of vitamin D in muscle strength and function are still being investigated. Studies on vitamin D pathway-related genotypes and muscle traits were exclusively focused on the VDR gene. Genomic and non-genomic pathways might explain the effects of vitamin D on muscles. While the genomic effect is mediated through the already mentioned 1,25D-VDR-RXR complex, the non-genomic effect involves intracellular calcium and phosphate homeostasis resulting from transcriptional regulation of specific proteins in organs such as intestines, bone, and parathyroid gland [125]. In this respect, it has been shown that muscle fibers of VDR-null mice were smaller, more variable in size, and accompanied by abnormal expression of myoregulatory transcription factors (myf5, myogenin, and E2A). Hence, it is believed that VDR may be involved in transcriptional down-regulation of these factors during muscle differentiation [137]. Whereas these implications and underlying biology are still being studied [138], this review shows rs2228570 and rs1544410 within the VDR gene to be the most frequently investigated polymorphisms also with respect to their impact on skeletal muscle traits. Interestingly, the identified studies showed non-conclusive results as, i.e., upper body strength (major allele-the beneficiary one) [106] was controversially affected by the FokI (rs2228570) genotype than the lower body strength (minor allele-the beneficiary one) [103,108,111,113]. Nevertheless, this demands further mechanistic investigations. For BsmI (rs1544410) genotype, the positive impact of major alleles in lower body muscles was confirmed in four studies [104,109,110,112]. A recent study, not being included in the systematic review focusing on further SNPs in the VDR gene (rs9729, rs17882106, rs7136534, rs11568820, rs10735810, rs4516035, and 11574024) did not reveal new findings, as neither muscle strength nor physical performance were associated to these genotypes [139]. To date, studies investigating the direct impact of vitamin D pathway-related genes (other than VDR gene) and muscle traits are still lacking.

Finally, this systematic review highlighted that there are promising candidate SNPs in vitamin D pathway-related genes that might impact vitamin D level and eventually muscle traits. However, it should be noted that heterogeneity among the selected studies represents a potential limitation, which also caused the decision to refrain from conducting a meta-analysis. Despite this limitation, the strength lies in the extensive information on individual SNPs in most of the relevant vitamin D pathway-related genes. To extract this detailed information from all included studies caused the rather long duration from the underlying systematic search to reporting the results. However, no conflicting results were found when comparing the outcomes of our study to recently published data [121,122,140,141,142,143,144,145,146,147,148,149]. The focus of recently published data remains in the same gene’s polymorphisms: GC, CYP2R1, VDR, CYP24A1, and CYP27B1. Except for these genes, Fediriko et al., 2019 and Jorde et al., 2019 identified potentially novel SNPs in vitamin D-related candidate genes (LRP2 and CUBN), but none of those were statistically significant [121,122].

## 5. Conclusions

To the best of our knowledge, this systematic review presents a very comprehensive update of the association of polymorphisms in vitamin D pathway-related genes with vitamin D status in healthy adults. While especially SNPs in the GC (rs2282679, rs4588, rs1155563, rs7041) and CYP2R1 genes (rs10741657, rs10766197, rs2060793) were confirmed to be associated with vitamin D levels in more than 50% of the respective studies, various muscle traits have been investigated only in relation to four different VDR polymorphisms (rs7975232, rs2228570, rs1544410, and rs731236) and outcomes remain inconclusive. Taken together, these data could be used in various ways: (1) to use the identified SNPs as candidate genes to be validated in further studies, (2) to identify individuals at potential risk, and (3) to optimize potential interventions with all these suggestions being important for precision nutrition. 

## Figures and Tables

**Table 1:** SNPs in vitamin D pathway-related genes and vitamin D status.

Name Gene/ID	Description	Aliases	Studies	SNPs Investigated in Association with Circulating Vitamin D Levels	
				Significant Associations	Non-Significant
GC ID:2638	GC, vitamin D binding protein	DBP, DBP-maf, DBP/GC, GRD3, Gc-MAF, GcMAF, HEL-S-51, VDB, VDBG, VDBP	68 different SNPs reported by 56 studies [26,27,28,29,30,31,32,33,34,35,36,37,38,39,40,41,42,43,44,45,46,47,48,49,50,51,52,53,54,55,56,57,58,59,60,61,62,63,64,65,66,67,68,69,70,71,72,73,74,75,76,77,78,79,80,81]	rs115316390, rs1155563 *, rs11939173, rs12512631 *, rs16846876 *, rs16847015, rs17467825 *, rs222020 *, rs222040, rs222054, rs2282679 *, rs2298849 *, rs2298850 *, rs3755967 *, rs4588 *, rs7041 *, rs705119, rs705120, rs842999, rs9016	rs10011000, rs10488854, rs12640179, rs12644050, rs13117483, rs1352841, rs1352843, rs1352844, rs1352845, rs1491709, rs1491710, rs1491711, rs1491718, rs1491719, rs1565572, rs16846912, rs16846943, rs16847019, rs16847024, rs16847028, rs16847039, rs16847050, rs17383291, rs17830803, rs1873590, rs188812, rs2070741, rs222003, rs222010, rs222014, rs222016, rs222017, rs222023, rs222029, rs222035, rs222043, rs222049, rs2276461, rs3733359, rs3737549, rs3775152, rs4694105, rs4752, rs6817912, rs6835052, rs705117, rs705124, rs843006
CYP2R1 ID:120227	cytochrome P450 family 2 subfamily R member 1	-	29 different SNPs reported by 41 studies [27,28,29,30,31,32,33,34,35,36,38,39,42,44,46,47,48,50,51,52,55,56,57,60,62,63,65,67,68,69,72,73,74,76,77,78,79,80,81,82,83]	rs10500804 *, rs10741657 *, rs10766197 *, rs10832306, rs11023374 *, rs11023380 * rs12794714 *, rs1562902 *, rs1993116 *, rs2060793 *, rs7116978, rs7935792	rs1037379, rs10832312, rs10832313, rs11023371, rs114050796, rs11819875, rs12418214, rs12419657, rs1496167, rs16930609, rs16930625, rs206793, rs7117967, rs7125348, rs7129781, rs7936142, rs952301
VDR ID:7421	vitamin D receptor	NR1I1, PPP1R163	111 different SNPs reported by 41 studies [27,28,29,30,33,37,41,42,46,47,50,52,55,56,57,60,61,62,63,65,67,71,74,75,77,78,84,85,86,87,88,89,90,91,92,93,94,95,96,97,98]	rs10783219 *, rs11568820, rs1544410 *, rs2228570 * (merged rs10735810), rs2239186 *, rs2408876, rs4516035, rs7139166, rs731236 *, rs7968585, rs7975232 *	rs10083198, rs10747524, rs10783215, rs10783218, rs10875693, rs10875694, rs10875695, rs10875702, rs10875712, rs11168264, rs11168266, rs11168268, rs11168275, rs11168277, rs11168287, rs11168288, rs11168292, rs11168302, rs11168314, rs11540149, rs11574024, rs11574026, rs11574027, rs11574038, rs11574042, rs11574044, rs11574065, rs11574077, rs11574110, rs11574113, rs11574138, rs11574141, rs11574143, rs11608702, rs11834903, rs12308082, rs12314197, rs12370156, rs12717991, rs12721364, rs12721365, rs12721370, rs1540339, rs17882106, rs1859281, rs1989969, rs2071358, rs2107301, rs2189480, rs2238135, rs2238136, rs2238138, rs2239179, rs2239180, rs2239181, rs2239182, rs2239184, rs2239185, rs2248098, rs2254210, rs2283342, rs2525044, rs2525045, rs2544027, rs2544038, rs2853559, rs2853560, rs2853564, rs3782905, rs3819545, rs3847987, rs4077869, rs4328262, rs4334089, rs4442605, rs4760648, rs4760655, rs4760658, rs4760674, rs6580642, rs7136534, rs7299460, rs7302038, rs7302235, rs7305032, rs7310552, rs7311030, rs739837, rs757343, rs7962898, rs7963776, rs7965281, rs7967152, rs7971418, rs7975128, rs7976091, rs881383, rs886441, rs9729, rs987849
CYP24A1 ID:1591	cytochrome P450 family 24 subfamily A member 1	CP24, CYP24, HCAI, HCINF1, P450-CC24	65 different SNPs reported by 31 studies [26,27,28,29,30,31,32,33,35,38,42,46,52,55,56,57,60,61,62,65,68,69,71,72,73,74,75,78,80,81,97]	rs17216707, rs2209314 *, rs2762939 *, rs2762941, rs6013897 *, rs73913757	rs11907350, rs13038432, rs1555439, rs1570669, rs1570670, rs17219315, rs1870969, rs2021940, rs2181874, rs2244719, rs2245153, rs2248137, rs2248359, rs2248461, rs2274130, rs2296239, rs2296241, rs2426496, rs2426498, rs2585413, rs2585422, rs2585423, rs2585428, rs2585439, rs2762926, rs2762929, rs2762932, rs35051736, rs3787555, rs3787557, rs3886163, rs4809957, rs4809958, rs4809959, rs4809960, rs6013905, rs6022990, rs6022999, rs6023005, rs6023009, rs6023012, rs6068810, rs6068812, rs6068816, rs6068824, rs6097797, rs6097801, rs6097805, rs6097809, rs6127112, rs6127119, rs73913755, rs751090, rs765058, rs765059, rs8124792, rs912505, rs927650, rs927651
DHCR7 ID:1717	7-dehydrocholesterol reductase	SLOS	25 different SNPs reported by 28 studies [27,28,29,31,32,33,35,36,38,39,42,44,46,51,52,55,56,57,60,67,69,72,73,74,77,78,81,99]	rs11603330, rs12785878 *, rs1790349 *	rs11233570, rs11234027, rs11606033, rs12419279, rs12800438, rs1540129, rs1540130, rs1790325, rs1790329, rs1790334, rs1790373, rs1792272, rs1792284, rs3794060, rs3829251, rs4316537, rs4944957, rs4945008, rs7122671, rs7944926, rs7950649, rs949178
CYP27B1 ID:1594	cytochrome P450 family 27 subfamily B member 1	CP2B, CYP1, CYP1alpha, CYP27B, P450c1, PDDR, VDD1, VDDR, VDDRI, VDR	15 different SNPs reported by 22 studies [26,27,28,30,33,37,41,42,46,52,55,56,60,62,65,74,75,77,80,81,97,100]	rs10877012 *	rs1021469, rs1048691, rs10877011, rs10877013, rs12368653, rs2269720, rs3782130, rs4646536, rs4646537, rs4760169, rs703842, rs8176344, rs8176345, −1077
CYP27A1 ID:1593	cytochrome P450 family 27 subfamily A member 1	CP27, CTX, CYP27	27 different SNPs reported by 7 studies [26,28,30,33,74,80,81]	--	rs116071925, rs11677711, rs12623740, rs12694443, rs12987009, rs12990447, rs13013510, rs13382651, rs17470271, rs4646535, rs4674338, rs4674344, rs4674345, rs6436084, rs6436094, rs645163, rs647952, rs6709815, rs6716642, rs6723334, rs6740004, rs6751527, rs7566656, rs7568196, rs7594289, rs7603709, rs933994
CASR ID:846	calcium-sensing receptor	CAR, EIG8, FHH, FIH, GPRC2A, HHC, HHC1, HYPOC1, NSHPT, PCAR1, hCasR	71 different SNPs reported by 6 studies [27,60,71,74,80,101]	rs17251221, rs1801725,	rs10222633, rs1042636, rs10934578, rs11715859, rs11716910, rs12485716, rs13093602, rs13324814, rs13327652, rs1354162, rs1393198, rs1501892, rs1501898, rs1501900, rs16832787, rs17203502, rs17203516, rs17282008, rs1801726, rs1814740, rs1847029, rs1973490, rs1979869, rs2036399, rs2134223, rs2134224, rs2173961, rs2202127, rs2221266, rs2270916, rs2279802, rs3749203, rs3749207, rs3792288, rs3792291, rs3804592, rs3804593, rs3804595, rs3845918, rs4677900, rs4678013, rs4678029, rs4678031, rs4678035, rs4678173, rs4678174, rs6438705, rs6438706, rs6438712, rs6764205, rs6764544, rs6768109, rs6776158, rs6799828, rs7614486, rs7617603, rs7628990, rs7635128, rs7639847, rs7644981, rs7647405, rs7648041, rs937626, rs9740, rs9820206, rs9826770, rs9866419, rs9875101, rs9875636
PTH ID: 5741	parathyroid hormone [Homo sapiens (human)]	PTH1	12 different SNPs reported by 6 studies [30,74,80,81,96,102]	rs10500783, rs1459015,	rs2593570, rs6254, rs6256, rs6264, rs694, rs10500784, rs177706, rs192802, rs3099597, rs751610
CYP3A4 ID:1576	cytochrome P450 family 3 subfamily A member 4	CP33, CP34, CYP3A, CYP3A3, CYPIIIA3, CYPIIIA4, HLP, NF-25, P450C3, P450PCN1	9 different SNPs reported by 5 studies [28,30,62,80,81]	rs2242480	rs12333983, rs2246709, rs2687116, rs2740574, rs35599367, rs3735451, rs4646437, rs6956344
RXRA ID: 6256	retinoid X receptor alpha	NR2B1	48 different SNPs reported by 3 studies [52,71,80]	rs11185644 *	rs1045570, rs10785870, rs10881577, rs10881578, rs10881580, rs10881582, rs10881583, rs11102986, rs11103473, rs11103482, rs11185647, rs11185659, rs12004589, rs12004786, rs1536475, rs1805348, rs1805352, rs12339187, rs3118523, rs3118526, rs3118536, rs3118570, rs3118571, rs3132294, rs3132296, rs3132299, rs3132300, rs34677682, rs35603635, rs3818738, rs3818739, rs3818740, rs4240705, rs4917347, rs4917352, rs4917353, rs4917354, rs6537944, rs7039190, rs7861779, rs7864987, rs7871655, rs842196, rs872298, rs877954, rs881657, rs914853
CUBN ID:8029	cubilin [Homo sapiens (human)]	IFCR, MGA1, gp280	14 different SNPs reported by 1 study [81]	--	rs10904881, rs11254370, rs1687705, rs1801222, rs1801223, rs1801224, rs1801225, rs1801231, rs1801232, rs1801234, rs1801241, rs2271462, rs3740165, rs703064
RXRB ID:6257	retinoid X receptor beta [Homo sapiens (human)]	DAUDI6, H-2RIIBP, NR2B2, RCoR-1	4 different SNPs reported by 1 study [81]	--	rs6531, rs2076310, rs3117040, rs9277935

SNPs-Single Nucleotide Polymorphisms, * significant associations with circulating vitamin D levels reported in more than one study.

**Table 2:** SNPs in the VDR genes and their association with various muscle traits.

SNP/Tra-ditional Name	Region, Reference	Participants (Number, Gender, Age)	Outcomes for Muscle Mass and Function	Main Findings
rs7975232/ ApaI	Tianjin, China [104]	*n* = 109 f (AA: 19.57 ± 0.53 y, Aa: 20.00 ± 1.20 y, aa: 19.89 ± 1.05 y)	Concentric and eccentric peak torque of knee extensors and flexors at 30°/s, 60°/s, and 180°/s; concentric peak torque of elbow extensors and flexors at 30°/s and 120°/s	aa + aA genotypes→higher knee extension peak torque at 120°s, higher elbow flexion at 120°/s and 30°/s than AA
	Fuki Prefecture, Japan [105]	*n* = 180 f; 60.1 ± 6.6 y	Handgrip strength; isokinetic concentric peak torque of knee extensors and flexors; isometric and isokinetic concentric and eccentric peak torque of trunk flexors and extensors	No significant differences in any muscle strength parameter between ApaI genotype groups (AA, Aa, aa) (data not shown in article)
	Northern Italy [106]	Centenarians (*n* = 102, 102.3 ± 0.3 y) versus septuagenarians (*n* = 163; 73.0 ± 0.6 y)	Handgrip strength	FF→significantly higher handgrip strength than Ff + ff
rs1544410/ BsmI	Sirente area, Italy [107]	*n* = 259 (87 m + 172 f); 85.0 ± 4.5 y	Handgrip strength, short physical performance battery (SPPB)	No significant differences between genotypes (BB, Bb, bb) in handgrip strength and SPPB score
	Baltimore, USA [108]	*n* = 864 (489 m + 375 f); 22–90 y;	Total and appendicular fat-free mass (DXA); handgrip strength; concentric peak torque of knee extensors at 30°/s and 180°/s; isometric peak torque at 120° and 140° knee ankle	No significant differences in fat-free mass Only modest differences in strength measurements: knee extensor isokinetic peak torque at 30°/s in females (bb > bB > BB)
	Northern Italy [106]	Centenarians (*n* = 102, 102.3 ± 0.3 y) versus septuagenarians (*n* = 163; 73.0 ± 0.6 y)	Handgrip strength	No significant differences in handgrip strength between genotypes
	Istanbul, Turkey [109]	*n* = 120 m; 69.0 ± 6.9 y	Fat-free mass (BIA); isokinetic peak torque of knee extensors, flexors at 60°/s	BB→higher knee extensor strength as compared to Bb + bb; No differences for flexors, muscle mass, and vitamin D level
	UK, Germany, France [110]	APUSS cohort (*n* = 3234 f; 54.3 ± 2.3 y); OPUS cohort (*n* = 1970 f; 66.9 ± 7.0 y)	Handgrip strength; chair rise test (difficulty and power with force plate); data only from OPUS cohort	BB + bB genotypes→higher max power, fewer difficulties to stand up from a chair No differences in handgrip strength
	London, UK [103]	COPD (*n* = 107; 75 m + 32 f; 63.5 ± 9.5y); age-matched controls (*n*= 104; 48 m + 56 f; 61.8 ± 8.5 y)	Handgrip strength; quadriceps strength, calculated as quadriceps maximum voluntary contraction force; fat-free mass (BIA)	No significant differences between genotypes for fat-free mass and strength measures
	Leuven, Belgium [111]	493(253 m (54.9 ± 10.2 y) + 240 f (41.5 ± 13.2 y))	Fat-free mass (estimated from skinfolds); handgrip strength; Isometric knee extension strength at 150°, 120°, and 90° knee ankle; Isometric knee flexion strength at 120°	No significant differences in any of the measured parameters [data not shown]
	Tianjin, China [104]	*n* = 109f (AA: 19.57 ± 0.53 y, Aa: 20.00 ± 1.20 y, aa: 19.89 ± 1.05 y)	Concentric and eccentric peak torque of knee extensors and flexors at 30°/s, 60°/s, and 180°/s; concentric peak torque of elbow extensors and flexors at 30°/s and 120°/s	BB + Bb genotypes→higher knee flexion peak torque at 180°/s than bb group No other differences
	Uppsala, Sweden [112]	*n* = 175 f; 29.6 ± 5.9	Handgrip strength; Isokinetic knee-flexion and extension strength at 90°/s; lean body mass (DXA)	BB→higher hamstring strength as compared to bb No differences in lean mass
	Monongahela Valley, USA [113]	*n* = 302 m; 58−93 y	Peak and average isometric quadriceps strength at 125° knee ankle; appendicular and total fat-free mass (DXA)	No differences in any measured parameter
	*n*/A [114]	*n* = 501 f; >70 y (mean age: 75 y)	isometric muscle strength of the quadriceps; handgrip strength	bb + Bb→higher quadriceps strength than BB in non-obese females, but not in obese females
rs2228570/ FokI (rs10735810)	Sirente area, Italy [107]	*n* = 259 (87 m + 172f ); 85.0 ± 4.5 y	Handgrip strength, short physical performance battery (SPPB)	No significant differences between genotypes (FF, Ff, ff) in handgrip strength and SPPB score
	Baltimore, USA [108]	*n* = 864 (489 m+ 375 f); 22–90 y;	Total and appendicular fat-free mass (DXA); handgrip strength; concentric peak torque of knee extensors at 30°/s and 180°/s; isometric peak torque at 120° and 140° knee ankle	No significant differences in fat-free mass; Males: no significant differences for any strength variable measured; Females: ff→higher isometric quadriceps strength (120°) in comparison to Ff and FF
	Northern Italy [106]	Centenarians (*n* = 102, 102.3 ± 0.3 y) versus septuagenarians (*n* = 163; 73.0 ± 0.6 y)	Handgrip strength	FF→significantly higher handgrip strength than Ff + ff
	Istanbul, Turkey [109]	*n* = 120 m; 69.0 ± 6.9 y	Fat-free mass (BIA); isokinetic peak torque of knee extensors, flexors at 60°/s	No significant differences between genotypes (FF, Ff, ff)
	UK, Germany, France [110]	APUSS cohort (*n* = 3234 f; 54.3 ± 2.3 y); OPUS cohort (*n* = 1970 f; 66.9 ± 7.0 y)	Handgrip strength; chair rise test (difficulty and power with force plate); data only from OPUS cohort	No significant differences between genotypes (FF, Ff, ff)
	London, UK [103]	cases with stable COPD (*n* = 107; 63.5 ± 9.5 y) and healthy age-matched controls (*n*= 104; 61.8 ± 8.5 y)	Handgrip strength; quadriceps strength, calculated as quadriceps maximum voluntary contraction force; fat-free mass (BIA)	FF→significantly lower quadriceps strength than Ff + ff
	Leuven, Belgium [111]	493(253 m (54.9 ± 10.2 y) + 240 f (41.5 ± 13.2 y))	Fat-free mass (estimated from skinfolds); handgrip strength; Maximal isometric knee extension strength at 150°, 120°, and 90° knee ankle; Maximal isometric knee flexion strength at 120° knee ankle;	Females: Ff→lower isometric knee extension strength at 120° and 90° than both FF and ff Males: no significant differences for any of the strength measurements
	Monongahela Valley, USA [113]	*n* = 302 m; 58–93 y	Peak and average isometric quadriceps strength at 125° knee ankle; appendicular and total fat-free mass (DXA)	FF→significantly lower appendicular and total fat-free mass than Ff and ff; FF→significantly lower peak and average isometric quadriceps strength than ff
rs731236/TaqI	Tianjin, China [104]	*n* = 109f (AA: 19.57 ± 0.53 y, Aa: 20.00 ± 1.20 y, aa: 19.89 ± 1.05 y)	Concentric and eccentric peak torque of knee extensors and flexors at 30°/s, 60°/s, and 180°/s; concentric peak torque of elbow extensors and flexors at 30°/s and 120°/s	No significant differences between genotypes (TT, Tt, tt)
	Fuki Prefecture, Japan [105]	*n* = 180 f; 60.1 ± 6.6 y	Handgrip strength; isokinetic concentric peak torque of knee extensors and flexors; isometric and isokinetic concentric and eccentric peak torque of trunk flexors and extensors	No significant differences between genotypes (TT, Tt, tt)
	Baltimore, USA [108]	*n* = 864 (489 m + 375 f); 22–90 y;	Total and appendicular fat-free mass (DXA); handgrip strength; concentric peak torque of knee extensors at 30°/s and 180°/s; isometric peak torque at 120° and 140° knee ankle	No significant differences in fat-free mass; Males: no significant differences for any strength variable measured; Females: ff→higher isometric quadriceps strength (120°) in comparison to Ff and FF
	Northern Italy [106]	Centenarians (*n* = 102, 102.3 ± 0.3 y) versus septuagenarians (*n* = 163; 73.0 ± 0.6 y)	Handgrip strength	FF→significantly higher handgrip strength than Ff + ff
	Istanbul, Turkey [109]	*n* = 120 m; 69.0 ± 6.9 y	Fat-free mass (BIA); isokinetic peak torque of knee extensors, flexors at 60°/s	No significant differences between genotypes (TT, Tt, tt)

n-number, y-years, f-females, m-males.

## Data Availability

The data presented in this study are available in Supplementary Table S1.

## Supplementary Materials

The following data are available online at www.mdpi.com/article/10.3390/nu13093109/s1, Table S1: SNPs overview.
